# Complex-Domain Semantic Segmentation of Spacecraft Directly from ISAR Echoes

**DOI:** 10.3390/s26134075

**Published:** 2026-06-26

**Authors:** Aoxiang Pan, Yonghua He, Yonggang Li, Jiahao Wang, Ruitao Shen, Weigang Zhu

**Affiliations:** 1 Space Engineering University, Beijing 101400, China; pax@hgd.edu.cn (A.P.); heyonghua1980@hgd.edu.cn (Y.H.); wjh_cwj@hgd.edu.cn (J.W.); srt_seu@hgd.edu.cn (R.S.); zwg@hgd.edu.cn (W.Z.); 2 National Key Lab of Space Target Awareness, Beijing 101400, China

**Keywords:** semantic segmentation, Inverse Synthetic Aperture Radar (ISAR), complex domain, automatic labeling, echoes

## Abstract

**Highlights:**

**What are the main findings?**

- We propose a complex-domain semantic segmentation framework OSS, which directly processes ISAR echoes to avoid scattering and phase information loss in traditional imaging pipelines.
- We design AIL, which enables automatic annotation of spacecraft masks based on ISAR scattering characteristics, effectively reducing manual labeling costs.

**What are the implications of the main findings?**

- We introduce a novel paradigm for spacecraft semantic segmentation, which effectively addresses the challenges of high annotation costs and information loss inherent in conventional imaging processes.
- We significantly reduce application costs by enabling automatic label generation and shortening the data processing chain.

**Abstract:**

Semantic segmentation technology based on Inverse Synthetic Aperture Radar (ISAR) images can provide crucial perception and analytical capabilities for intelligent safety maintenance of on-orbit spacecraft. However, conventional semantic segmentation methods suffer from three main limitations: firstly, the lack of modeling for radar physical characteristics in the “image first, segment later” pipeline leads to loss of scattering information and phase details; secondly, reliance on extensive pixel-level manual annotation increases application costs; thirdly, ineffective utilization of spacecraft structural priors fails to guide networks to focus on the main body and edges of spacecraft segmentation. To address these issues, this paper proposes a complex-domain semantic segmentation framework named One-Stop Segmentation (OSS) based on ISAR echoes. The framework incorporates two innovative modules: an Automatic ISAR Labeling (AIL) method designed based on ISAR scattering characteristics to generate labels corresponding to ISAR echoes, and a complex-domain semantic segmentation network named One-Stop Segmentation Network (OSSNet) that performs semantic segmentation directly on echoes, avoiding information loss from imaging while shortening the data processing chain. Core contributions of OSSNet include: (1) a Domain Alignment Module (DAM) to effectively mitigate domain mismatch caused by data distribution differences between raw echo signals and labels; (2) a Multi-Perspective Attention (MPA) framework incorporating a Sliding Correlation Attention (SCA) module and a Subdomain Balanced Attention (SBA) module, lever-aging spacecraft structural priors to guide the network’s focus on main structures and edge details from complementary perspectives, significantly improving segmentation ac-curacy. Experimental results on a simulated ground-based radar dataset demonstrate that the proposed OSS framework achieves a mean Intersection over Union (mIoU) of 92.13% and a mean F1-score of 95.75% in ISAR spacecraft semantic segmentation tasks, outperforming existing methods.

## 1. Introduction

In recent years, the rapid development of global civil space missions has led to a significant increase in the number of on-orbit satellites. Concurrently, the risk of space collisions continues to rise, with occasional on-orbit impact events that can cause structural damage to satellite platforms or key payloads, severely threatening the safe on-orbit operation and long-term service capability of spacecraft. Against this backdrop, achieving fine-grained perception and state identification of space targets has become a core issue urgently requiring solution in the field of on-orbit spacecraft safety maintenance. Inverse Synthetic Aperture Radar (ISAR) [1], with its advantages of all-weather and all-day capabilities, long operating range, and high resolution, has emerged as a crucial technological means for maintaining the safe on-orbit operation of space targets. ISAR-based spacecraft semantic segmentation can not only accurately estimate component dimensions and identify main structures [2] but also further assess their operational status and enable precise spacecraft positioning [3]. This provides substantial information support for collision damage assessment and fault diagnosis.

Traditional semantic segmentation methods often struggle to achieve precise segmentation of complex targets. With the advancement of deep learning, its powerful nonlinear modeling and feature extraction capabilities have provided new solutions for ISAR semantic segmentation. As one of the core tasks in computer vision, deep learning-based semantic segmentation has been widely applied in fields such as object recognition [4], remote sensing interpretation [5], and pose estimation [6]. These methods employ end-to-end pixel-level classification and leverage large-scale data training to automatically learn and extract high-level semantic features from images, thereby significantly distinguishing between different targets and backgrounds. Compared to traditional algorithms, deep neural networks can fully utilize high-order semantic information, maintaining high segmentation accuracy even in complex environments and with target variations [7].

Currently, numerous scholars have conducted extensive research on ISAR semantic segmentation. Zhu et al. integrated semantic segmentation with mask matching [8] to enhance the model’s generalization capability. Kou et al. introduced contrastive learning and a non-local U-Net [9], first using binary semantic labels for coarse segmentation of the target contour, then employing a non-local self-attention mechanism with global perception to guide the model to focus on the structural symmetry of ISAR images, thereby improving segmentation accuracy by enhancing feature discriminability. Coe et al. improved the Statistical Region Merging (SRM) algorithm [10], adapting its original three-channel optical-image mechanism to single-channel ISAR intensity data for effective image segmentation. Zhong et al. proposed the Scattering Characteristics Guided Network (SCGN) [11], which incorporates scattering features of various components into the mask segmentation process and models long-range semantic dependencies through a spatial attention mechanism to achieve fine-grained component segmentation. Ju et al. leveraged the inherent multi-scale stochastic structures of ISAR images to propose a Spatially Variant Mixture Multiscale Autoregressive (SVMMAR) model [12] for efficient ISAR image segmentation. Zheng et al. proposed an unsupervised segmentation method for ISAR images [13], using a set of multi-scale autoregressive models to characterize the inherent multi-resolution stochastic structures in ISAR images for reliable image segmentation. Wang et al. proposed a segmentation concept based on the CLEAN algorithm [14], designing an adaptive region-growing method to segment the main body, with a focus on analyzing and solving the threshold setting problem in region-growing methods. It is noteworthy that most existing research on ISAR semantic segmentation predominantly leverages amplitude information [15], often neglecting the rich phase information inherent in radar signals.

In parallel with the ISAR-specific methods discussed above, attention mechanisms have become a key component of modern semantic segmentation architectures. Representative designs include the Squeeze-and-Excitation Network (SE-Net) [16] for channel-wise recalibration, the Convolutional Block Attention Module (CBAM) [17] for joint channel-spatial attention, non-local networks [18] for long-range contextual aggregation, and wavelet-based attention mechanisms [19] for multi-frequency feature decomposition. Despite their success in natural image segmentation, these mechanisms are fundamentally designed for real-valued feature representations and have not been adapted to exploit the complex-valued nature of radar echoes.

Although the aforementioned ISAR-specific methods and general attention mechanisms have advanced segmentation performance in their respective settings, their direct application to complex-domain spacecraft segmentation is hindered by three limitations. First, the predominant “image-first, segment-later” paradigm relies on complex imaging that projects complex-valued echoes onto real-valued amplitude images, irreversibly discarding phase information critical for characterizing target characteristics. Existing attention mechanisms, being architected for real-valued features, inherit rather than resolve this information bottleneck. Although Li et al. attempted to incorporate echo-domain features for few-shot segmentation [20], this was limited to a preliminary real-domain mapping without constructing a full complex-domain network. Second, standard self-attention computes pairwise feature correlations globally and uniformly. In ISAR data, this biases attention toward isolated strong scattering points while suppressing the weaker yet structurally continuous responses that delineate component boundaries. Meanwhile, these data-driven mechanisms lack embedded priors about spacecraft geometry and radar scattering physics which are essential for distinguishing genuine structural edges from coherent sidelobe artifacts. Third, label acquisition remains dependent on costly manual annotation [21], and the imaging preprocessing step introduces both information loss and computational latency unsuitable for time-sensitive space situational awareness tasks. These factors collectively hinder the model’s capability to achieve high-precision segmentation of space targets.

To address the aforementioned issues, this paper proposes an end-to-end complex-domain ISAR echo semantic segmentation framework named One-stop Semantic Segmentation (OSS). The OSS framework comprises an automatic labeling method Auto ISAR Labelling (AIL) and a complex-domain segmentation network One-stop Semantic Segmentation Network (OSSNet), achieving direct processing from echo signals to semantic segmentation results. Experimental results demonstrate that the proposed method significantly outperforms existing ISAR semantic segmentation approaches. The main contributions of this paper are summarized as follows:

- An Automatic ISAR Labeling (AIL) method that generates semantic masks from complex ISAR images using multi-scale gradient fusion, eliminating manual annotation costs.
- An end-to-end One-Stop Segmentation Network (OSSNet) that directly maps raw ISAR echoes to segmentation masks, bypassing traditional imaging pipelines.
- A Domain Alignment Module (DAM) that bridges the data distribution gap between raw echoes and image-domain labels via learnable domain transformation.
- A Multi-Perspective Attention (MPA) framework (SCA + SBA) that integrates spacecraft structural priors with full-dimensional scattering characteristics, enabling precise segmentation of main structures and edges.

## 2. Proposed Method

### 2.1. OSS-Based ISAR Semantic Segmentation

High-precision ISAR semantic segmentation is crucial for on-orbit spacecraft maintenance. However, its advancement faces constraints due to the limited availability of high-quality annotated data and insufficient data utilization. Furthermore, traditional methods often struggle to address challenges such as discontinuous target scattering points and sidelobe interference in ISAR imagery, while generally neglecting phase information and prior structural knowledge of spacecraft. These limitations hinder effective modeling of complete physical scattering characteristics, consequently restricting segmentation accuracy. To overcome these issues, this paper proposes an end-to-end complex-domain semantic segmentation framework termed OSS, which achieves direct signal-to-segmentation mapping in the raw echo domain, thereby circumventing information loss inherent in conventional imaging-segmentation cascade processing. The OSS framework comprises two core innovative modules: the AIL method for automatic high-quality mask generation based on ISAR scattering characteristics, and the segmentation network OSSNet.

**Figure 1:**
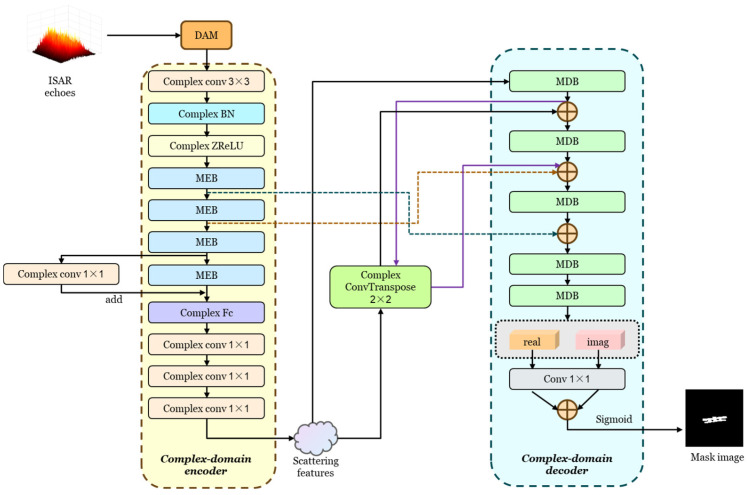
Architecture of the proposed OSSNet, showing the data flow from raw ISAR echo input through DAM, multi-branch encoder with MPA, to the decoder with skip connections, and finally the output segmentation mask.

**Figure 2:**
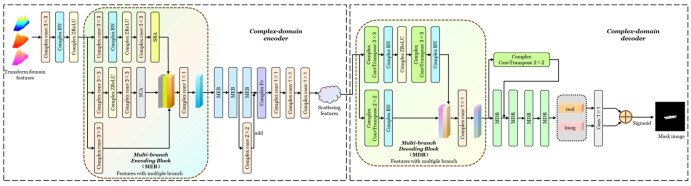
Detailed architecture: (**left**) Complex-domain encoder; (**right**) Complex-domain decoder. The cloud-shaped pattern therein is an abstract representation of the scattering features obtained by the encoder.

Figure 1 illustrates the overall framework of OSSNet, while Figure 2 depicts the architectures of the encoder and decoder, respectively. The network takes raw ISAR echo signals as input. First, the DAM applies a learnable orthogonal transformation to the echo data, mapping the original signal domain to a more discriminative feature domain, effectively resolving the domain mismatch between echoes and mask labels. The transformed features are then fed into a multi-branch encoder integrated with the MPA framework. This encoder extracts scattering features at different hierarchical levels through parallel paths, and incorporates the SBA and SCA modules to enhance the representation capability for spacecraft main structures and detailed features. The decoder adopts a multi-branch structure without attention mechanisms, progressively performing upsampling and leveraging skip connections to fuse multi-scale features from the encoder. This design fully utilizes shallow high-resolution information to improve spatial localization accuracy, while combining deep semantic features for precise classification, ultimately outputting high-precision semantic masks. The AIL method is detailed in Section 2.2, and implementation specifics of OSSNet are provided in Section 2.3, Section 2.4 and Section 2.5.

### 2.2. Automatic ISAR Labeling (AIL)

In the field of ISAR semantic segmentation, target segmentation masks are typically obtained through manual annotation. However, manual annotation methods are often accompanied by substantial labor and time costs. Furthermore, the high structural complexity of spacecraft targets, combined with characteristics such as discontinuous scattering point distribution, significant intensity variations in scattering points at different positions, and edge blurring, makes it difficult for manual annotation to meet precision requirements. Therefore, achieving automatic and accurate data annotation based on ISAR scattering characteristics has become a critical issue requiring urgent resolution in this field.

**Figure 3:**
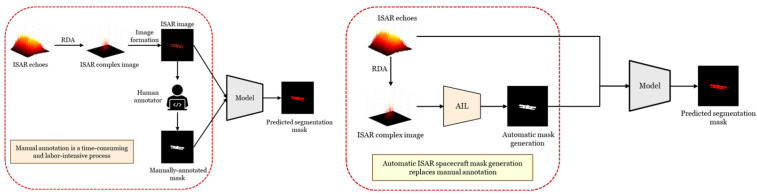
Comparison of annotation pipelines: (**left**) Traditional manual annotation workflow, requiring imaging operations and expert labeling; (**right**) Proposed AIL method, which directly generates masks from complex ISAR images via multi-scale gradient fusion and morphological post-processing.

To address these challenges, this paper proposes an automatic labeling method called AIL based on ISAR scattering characteristics. The core of AIL lies in constructing multi-scale fused complex gradient images: it enhances edge completeness by capturing multi-resolution edge features and employs a multi-scale feature weighting fusion mechanism to prevent small-scale details from being overwhelmed by large-scale structures. Furthermore, morphological post-processing is introduced to improve the geometric accuracy and topological completeness of the segmentation masks. This method essentially represents the mapping of target electromagnetic scattering physical characteristics into image space, providing theoretical support for generating high-precision spacecraft segmentation masks. Unlike traditional annotation workflows—where manual labeling is performed on ISAR images after imaging transformation and imaging compensation, as shown in Figure 3 (left)—AIL directly achieves automated labeling in the complex ISAR image domain according to its electromagnetic scattering characteristics, as illustrated in Figure 3 (right).

In ISAR imaging, the physical edges of spacecraft produce significant gradient responses due to variations in electromagnetic scattering characteristics. Specifically, metal structural components such as the spacecraft main body and solar panels form strong scattering centers in complex ISAR images, where the complex signals undergo sharp variations at boundary regions, resulting in high gradient magnitudes. Structural junction areas exhibit medium-to-high gradient magnitudes due to scattering discontinuity, while background regions generally demonstrate low gradient magnitudes. This characteristic provides a physical basis for distinguishing target structures from background regions using gradient magnitude thresholds, thereby enabling the generation of effective target segmentation masks. $Region\;of\;Interest=\left\{(x,y)|\left\|\nabla G(x,y)\right\|\geq \tau \right\}$, where τ is the threshold.

Inspired by the Laplacian pyramid method [22], a K-layer Gaussian pyramid is first constructed through Gaussian smoothing filtering and progressive downsampling, thereby forming a multi-scale complex image space. Let the complex-valued image matrix at the k-th layer be denoted as $G_{k}\in {\mathbb{C}}^{M_{k}\times N_{k}}$. Then, $G_{k}(x,y)=R_{k}(x,y)+I_{k}(x,y)\cdot j$, where j is the imaginary unit.

The complex convolution operation is separable, allowing convolution operations to be performed separately on the real and imaginary parts:

(1) $$\left\{\begin{array}{l} R_{k-1}^{smooth}(x,y)=\sum_{i=-r}^{r}\sum_{j=-r}^{r}R_{k-1}(x+i,y+j)\cdot g_{\sigma }(i,j)\\ I_{k-1}^{smooth}(x,y)=\sum_{i=-r}^{r}\sum_{j=-r}^{r}I_{k-1}(x+i,y+j)\cdot g_{\sigma }(i,j) \end{array}\right.$$

where $g_{\sigma }(x,y)$ denotes the discrete Gaussian kernel function, and σ denotes the standard deviation, which varies across pyramid levels: $\sigma_{k}=\sigma_{1}\cdot 2^{k-1}$, $r=\left\lfloor 3\sigma \right\rfloor$.

Define the complex downsampling operator $D:{\mathbb{C}}^{X\times Y}\to {\mathbb{C}}^{\left\lfloor X/2\right\rfloor \times \left\lfloor Y/2\right\rfloor }$, D(G)(x,y)=G(2x−1,2y−1). Namely,

(2) $$\left\{\begin{array}{l} R_{k}(m,n)=R_{k-1}^{smooth}(2m-1,2n-1)\\ I_{k}(m,n)=I_{k-1}^{smooth}(2m-1,2n-1) \end{array}\right.$$

where $m=1,2,\ldots ,\left\lfloor M/2\right\rfloor ,n=1,2,\ldots ,\left\lfloor N/2\right\rfloor$.

The recurrence formula for complex images at each layer is as follows:

(3) $$\left\{\begin{array}{l} G_{k}=D\left(\left[\begin{array}{l} R_{k-1}*g_{\sigma_{k}}\\ I_{k-1}*g_{\sigma_{k}} \end{array}\right]\right)\\ \sigma_{k}=\sigma_{1}\cdot 2^{k-1} \end{array}\right.$$

where ∗ denotes the two-dimensional Gaussian convolution operation, with k=2,3…,K.

Subsequently, multi-scale gradient magnitude calculation is performed. Starting from the original-size complex image $G_{1}$, the finite difference method is used to calculate its real part gradient and imaginary part gradient along the horizontal and vertical directions of the image, thus obtaining the original-scale gradient magnitude image $M_{1}$; for the k-th layer (k≥2) complex image matrix $G_{k}$, its gradient calculation uses the Sobel operator. Using its horizontal and vertical convolution kernels, the gradient responses of the image in two orthogonal directions are calculated respectively, thus obtaining the k-th layer gradient magnitude image $M_{k}$.

(4) $$\left\{\begin{array}{l} M_{1}=\sqrt{{\left|\nabla_{x}R_{1}+j\nabla_{x}I_{1}\right|}^{2}+{\left|\nabla_{y}R_{1}+j\nabla_{y}I_{1}\right|}^{2}}\\ M_{k}=\sqrt{{\left|\nabla_{x}^{sobel}R_{k}+j\nabla_{x}^{sobel}I_{k}\right|}^{2}+{\left|\nabla_{y}^{sobel}R_{k}+j\nabla_{y}^{sobel}I_{k}\right|}^{2}} \end{array}\right.$$

where ∇ denotes the gradient computation.

The BiCubic interpolation algorithm [23] is employed to upsample the k-th layer gradient magnitude image to the original scale image resolution, i.e., $M_{k}\in {\mathbb{R}}^{\frac{M_{1}}{2^{k-1}}\times \frac{N_{1}}{2^{k-1}}}$⇒ $M_{k}\in {\mathbb{R}}^{M_{1}\times N_{1}}$.

Weighted fusion is performed on the multi-scale gradient magnitude images:

(5) $$M_{final}=\sum_{k=1}^{K}w_{k}M_{k},w_{k}=\left\{\begin{array}{l} 1,k=1\\ \frac{1}{2k},k=2,3,\ldots ,K \end{array}\right.$$

At the original scale level, the second-order accuracy characteristic of the central difference algorithm: $\nabla_{x}A=\frac{A(x,y+1)-A(x,y-1)}{2\Delta y}+O(\Delta y^{2})$ enables sub-pixel localization of spacecraft edges, ensuring clear segmentation boundaries. Meanwhile, the Sobel operator provides high computational efficiency while maintaining linear characteristics by combining smoothing and differentiation operations. By integrating these two gradient calculation methods in multi-scale space, the localization accuracy of spacecraft structural edges can be ensured while maintaining low computational complexity.

Finally, morphological post-processing is applied to the fused multi-scale complex gradient image to generate the final segmentation mask. The closing operation is first applied to effectively fill internal cavities caused by weak scattering regions and bridge edge discontinuities; subsequently, the opening operation is employed to smooth boundaries and suppress isolated artifact points.

The complete workflow of the AIL method is shown in Algorithm 1.
**Algorithm 1:** Auto ISAR Labelling**Input:** $\mathrm{Complex}\;\mathrm{image}\;\mathrm{matrix}\;G_{1}\in {\mathbb{C}}^{M\times N}$ **Output:**$\;\mathrm{Segmentation}\;\mathrm{mask}\;S\in {\mathbb{R}}^{M\times N}$ **Part 1:** Gaussian pyramid construction  **for** i={2,3,…,K} **do**    $G_{i}=reduce(G_{i-1})$, where reduce(⋅) represents Gaussian smoothing and downsampling  **end** **Part 2:** Multi-scale complex gradient fusion  $M_{1}=\left\|\nabla G_{1}\right\|$  $M=M_{1}$  **for** i={2,3,…,K} **do**   $M_{i}=resize(\left\|\nabla_{Sobel}G_{i}\right\|)$, where resize(⋅) represents Bicubic interpolation   $M=M+\frac{1}{2i}M_{i}$  **end** **Part 3:** Morphological processing  $Z=\left\{\begin{array}{l} 0,M\leq \tau \\ 1,M>\tau \end{array}\right.$, where τ represents Threshold  Z=closing(Z,rect), where closing represents closing operation  S=opening(Z,disk), where opening represents opening operation **return** S


### 2.3. Domain Alignment Module (DAM)

Conventional ISAR segmentation masks are generated based on target-background edge localization in the image domain, sharing spatial domain characteristics with ISAR imagery. Consequently, neural networks can directly learn pixel spatial relationships to produce masks. However, ISAR echoes require complex imaging transformations to form images, creating significant data distribution differences between the echo domain and mask domain—a phenomenon termed “domain mismatch.” Directly transferring image segmentation algorithms to the echo domain leads to model incompatibility due to this domain mismatch, consequently limiting segmentation accuracy. Furthermore, while ISAR images essentially represent mappings of echo data into the pixel domain, existing image-based segmentation methods utilize only amplitude information while discarding the phase component, resulting in insufficient modeling of original scattering characteristics. Since both amplitude and phase in the echoes carry target scattering properties, the loss of phase information causes critical physical features to be overlooked, thereby constraining further improvements in segmentation accuracy.

**Figure 4:**
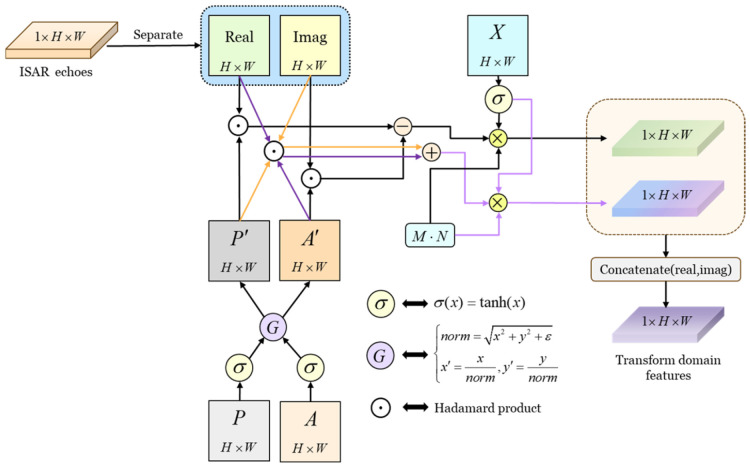
Structure of the DAM. The statement “c=Concatenate(a,b)” means that c=a+b⋅j.

To address the aforementioned challenges of echo-mask domain mismatch and incomplete utilization of physical scattering characteristics, this paper proposes a DAM, whose structure is illustrated in Figure 4. The module constructs an adaptive feature transformation function through learnable parameter matrices {P,A,X} and learnable parameters {M,N}, enabling dynamic mapping of input echo data into a feature space compatible with the mask domain. This effectively bridges the data distribution gap between the echo and mask domains while significantly accelerating model convergence by compressing the solution space, ultimately improving segmentation accuracy and enhancing model generalization capability.

Let the input of DAM be the ISAR echo $E\in {\mathbb{C}}^{1\times H\times W}$, where dimensions 1,H,W correspond to the number of channels, azimuth samples, and range samples, respectively. The implementation workflow of DAM is as follows: First, by introducing learnable transformation matrices $\left\{P,A,X\in {\mathbb{R}}^{H\times W}\right\}$, a feature space mapping is constructed. The tanh(⋅) function is employed to constrain matrix values within the range (−1,1). This range normalization eliminates amplitude scale disparities to enhance optimization stability, while the sign preservation mechanism maintains the discriminability of phase relationships. Ultimately, a robust basis for feature representation is established in the transformed domain. The corresponding mathematical operations are expressed as follows:

(6) $$\tilde{P},\tilde{A},X^{\prime }=\tanh(P,A,X)$$

Based on this, unit modulus constraints are applied to the transformation matrices P and A, as shown in (7), forcing the basis vectors to lie on the unit circle in the complex plane to maintain the numerical stability of the complex transformation. Building upon this, a learnable rotation transformation is performed on the input echoes. This process extracts global features while preserving the signal’s phase characteristics. This design enables the model to adaptively learn the optimal complex basis space, achieving optimization of feature representation.

(7) $$\left[\begin{array}{c} P^{\prime }\\ A^{\prime } \end{array}\right]=\frac{1}{\sqrt{\tilde{P}⊙\tilde{P}+\tilde{A}⊙\tilde{A}+\varepsilon }}⊙\left[\begin{array}{c} \tilde{P}\\ \tilde{A} \end{array}\right]$$

where ⊙ denotes the Hadamard product, and ε is an extremely small positive number used to maintain numerical stability.

The ISAR echo is intrinsically a single-channel complex-valued matrix $E\in {\mathbb{C}}^{1\times H\times W}$. After channel dimension compression, $E\in {\mathbb{C}}^{H\times W}$ can be decomposed into real and imaginary components, i.e., E=R+Ij, where $R,I\in {\mathbb{R}}^{H\times W}$. Through adaptive transformation, the features of the real and imaginary parts are recombined and reconstructed, achieving an optimized representation of complex-domain information, as shown in (8).

(8) $$\left[\begin{array}{c} R_{t}\\ I_{t} \end{array}\right]=\sum_{i=1}^{H}\sum_{j=1}^{W}\underset{\mathrm{Rotation}\;\mathrm{matrix}}{\underset{︸}{\left[\begin{array}{cc} {P^{\prime }}_{ij} & -{A^{\prime }}_{ij}\\ {A^{\prime }}_{ij} & {P^{\prime }}_{ij} \end{array}\right]}}\left[\begin{array}{c} R_{ij}\\ I_{ij} \end{array}\right]$$

where Let $Q_{ij}=\left[\begin{array}{cc} {P^{\prime }}_{ij} & -{A^{\prime }}_{ij}\\ {A^{\prime }}_{ij} & {P^{\prime }}_{ij} \end{array}\right]$, for every (i,j):

(9) $$Q_{ij}^{T}Q_{ij}=Q_{ij}Q_{ij}^{T}=\left[\begin{array}{cc} {P^{\prime }}_{ij}^{2}+{A^{\prime }}_{ij}^{2} & 0\\ 0 & {P^{\prime }}_{ij}^{2}+{A^{\prime }}_{ij}^{2} \end{array}\right]$$

As derived from (7),

(10) $$\left\{\begin{array}{l} {P^{\prime }}_{ij}^{2}+{A^{\prime }}_{ij}^{2}=1\\ Q_{ij}^{T}Q_{ij}=Q_{ij}Q_{ij}^{T}=I_{2} \end{array}\right.$$

where $I_{2}$ denotes the identity matrix, and matrix $Q_{ij}$ is an orthogonal matrix. Hence each position-wise complex weight $W_{ij}=P_{ij}+jA_{ij}$ lies on the unit circle in the complex plane, and the local transformation constitutes a rotation in ${\mathbb{R}}^{2}$.

Simultaneously, feature space modulation is implemented through the learnable spatial weight matrix X, enhancing the model’s feature selection capability for critical regions. Learnable scaling factors {M,N∈ℝ} are introduced to dynamically adjust the feature amplitudes in the transformed domain, thereby maintaining numerical stability.

(11) $$\left[\begin{array}{c} R^{\prime }\\ I^{\prime } \end{array}\right]=M\cdot N\cdot \left[\begin{array}{c} R_{t}\\ I_{t} \end{array}\right]\cdot X^{\prime }$$

Finally, adjust the dimensions of the transformed features, i.e., $R^{\prime },I^{\prime }\in {\mathbb{R}}^{H\times W}\Rightarrow R^{\prime },I^{\prime }\in {\mathbb{R}}^{1\times H\times W}$, and combine the transformed complex features to obtain the transformed domain feature $E^{\prime }=R^{\prime }+I^{\prime }j$.

In the DAM, the learnable matrices P, A, and X serve distinct roles. P and A are normalized element-wise to form complex weights of unit modulus, which apply a pure phase rotation to each spatial position of the input echo. This step is locally strictly orthogonal—it preserves the instantaneous power of every resolution cell and avoids information loss or distortion in the phase component. X, by contrast, operates after the global summation: it modulates the compressed scalar back to the original spatial size and provides location-selective re-scaling. Because the overall transformation involves cross-spatial summation followed by an outer-product reconstruction, the equivalent mapping matrix is not an orthogonal matrix. Therefore, DAM as a whole does not constitute a strictly orthogonal transformation.

Nevertheless, the locally orthogonal design remains crucial. Constraining complex weights to the unit circle bounds the dynamic range of the parameters and prevents unbounded amplification during training, which markedly improves the numerical stability of gradient propagation and accelerates convergence. More importantly, the element-wise rotation retains the complete phase information of each echo sample, providing a stable representation that preserves full scattering characteristics for the subsequent encoder.

### 2.4. Complex-Domain Encoder

Radar-target relative aspect variations induce amplitude and phase perturbations in echoes, which manifest as edge blurring and strong scattering point sidelobe interference in ISAR imagery. These artifacts fundamentally originate from distortions in scattering characteristics within the echo domain. Consequently, integrating spacecraft rigid-body structural properties with ISAR scattering mechanisms to extract spatially discriminative, efficient, and robust features from echoes constitutes a critical challenge for enhancing semantic segmentation accuracy.

To address the aforementioned challenges and construct discriminative feature representations for precise semantic segmentation, this paper proposes a complex-domain encoder. The network first employs a cascaded structure of 3 × 3 Complex-domain Convolutional (CConv) layer, Complex-domain Batch Normalization (CBN), and Complex-domain ReLU (CReLU) activation function [24] to extract key local features in the transformed domain while enhancing nonlinear modeling capabilities. The features are subsequently fed into a Multi-branch Encoding Module (MEB) to hierarchically capture multi-scale scattering characteristics, where shallow layers preserve detailed information while deeper layers encapsulate high-level semantic information. The processed features then pass through a Complex-domain Fully connected layer (CFc) for global feature integration and compression. Finally, a 1 × 1 CConv is applied during the encoding stage to enrich feature representation, outputting a scattering point feature representation fused with global semantics. The complex-domain encoder is primarily constructed through the synergistic integration of CConv/CBN/CReLU, MEB, and CFc components.

The MEB adopts a multi-branch feature fusion architecture, significantly enhancing the diversity and discriminability of scattering features through complementary multi-perspective feature integration within hierarchical layers. The MPA framework proposes dual modules—SCA and SBA—capable of dynamically balancing the weights of amplitude and phase subdomains in foreground and background regions. These modules guide the network to focus on critical features of main structures and edge details across different dimensions, thereby improving semantic segmentation accuracy and model interpretability.

The MEB adopts a triple-branch heterogeneous feature fusion architecture. Through parallel extraction of original transformed features, correlation attention-enhanced features, and subdomain attention-enhanced features, complementary fusion of scattering representations is achieved. This design breaks through the limitations of single-path representations, effectively preserving the spatial continuity of target structures while ensuring edge localization accuracy, all while enhancing feature diversity.

Let the input feature of MEB be $E_{in}\in {\mathbb{C}}^{C_{in}\times H_{in}\times W_{in}}$, where $C_{in},H_{in},W_{in}$ represent the input channel number, feature map height, and feature map width of MEB, respectively. The original feature transformation branch $B_{r}$ consists of a single 3 × 3 CConv layer, expressed as:

(12) $$E_{origin}=B_{r}\left(E_{in}\right)$$

The correlation attention feature enhancement branch $B_{c}$ consists of two 3 × 3 CConv layers, one CReLU activation, and one SCA module. Its operational workflow is expressed as:

(13) $$E_{correlation}=B_{c}\left(E_{in}\right)$$

The subdomain attention feature enhancement branch $B_{s}$ consists of two 3 × 3 CConv layers, one CBN layer, one CReLU activation, and one SBA module. Its computational flow is expressed as:

(14) $$E_{subdomain}=B_{s}\left(E_{in}\right)$$

The triple-branch heterogeneous features are concatenated along the channel dimension to generate the combined features $E_{tri}$, which are then processed through a 1 × 1 CConv layer for cross-channel feature fusion and dimensional compression, outputting the optimized representation $E_{out}$ of MEB.

(15) $$\left\{\begin{array}{l} E_{tri}=concatenate(E_{origin},E_{correlation},E_{subdomain})\\ E_{out}=Complex\text{\_}Conv(E_{tri}) \end{array}\right.$$

#### 2.4.1. Sliding Correlation Attention (SCA)

On-orbit spacecraft exhibit highly regular geometric structures, with key components arranged in strongly symmetric patterns [9]. The complex interconnections between components and uneven distribution of scattering point intensities cause models to perceive the overall spacecraft structure weakly and blur boundaries.

**Figure 5:**
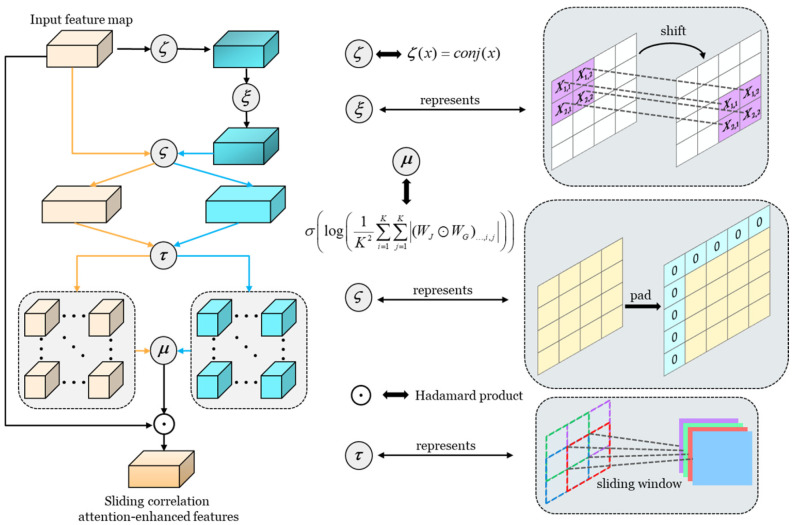
Structure of the SCA, where conj(⋅) denotes the conjugate operation on complex feature maps.

To address the aforementioned issues of structural awareness weakening and edge localization inaccuracy, inspired by the autocorrelation function—which, by the Wiener-Khinchin theorem, relates the autocorrelation of a signal to its power spectral density and thus provides a principled means of detecting periodic or symmetric structural patterns—this paper proposes the SCA module. This module employs a local autocorrelation operator to compute the spatial structural correlations of feature maps, generating a structural spatial attention map to selectively enhance feature responses of key components. This enables the model to precisely focus on highly correlated regions to extract structural main bodies and edge features, significantly improving structural perception accuracy. The structure of the SCA module is shown in Figure 5.

First, let the input feature map of the SCA module be $J\in {\mathbb{C}}^{C\times H\times W}$, and construct its complex conjugate feature map $\bar{J}\in {\mathbb{C}}^{C\times H\times W}$.

Next, generate the shifted feature map $G_{c,i,j}={\bar{J}}_{c,f_{h}(i),f_{w}(j)}$. The height dimension shift mapping relationship is:

(16) $$f_{h}(i)=\left\{\begin{array}{ll} i-win\text{\_}h & if\;i\geq win\text{\_}h\\ H+(i-win\text{\_}h) & if\;i<win\text{\_}h \end{array}\right.$$

The width dimension shift mapping relationship is:

(17) $$f_{w}(j)=\left\{\begin{array}{ll} j-win\text{\_}w & if\;j\geq win\text{\_}w\\ W+(j-win\text{\_}w) & if\;j<win\text{\_}w \end{array}\right.$$

where win_h and win_w represent the height shift step and width shift step, respectively.

Next, sliding window extraction is performed. To maintain consistent dimensions between the output and input feature maps, zero-padding is applied on all sides to both the input feature map and the shifted feature map. Let the padding length on each side be l, then $l=\left\lfloor \frac{K}{2}\right\rfloor$, where K is the size of the sliding window and $\left\lfloor \cdot \right\rfloor$ denotes the floor function. After padding, the features become $J_{pad},G_{pad}\in {\mathbb{C}}^{C\times (H+2l)\times (W+2l)}$. In this paper, the height and width dimensions of the feature maps are identical, i.e., H=W. A square sliding window of size K×K is used with a stride of 1, so the padding length is the same for both the height and width dimensions, both being l. Sliding window decomposition is then performed, resulting in $W_{J},W_{G}\in {\mathbb{C}}^{C\times K\times K}$, meaning each window corresponds to a spatial location in the original feature map. The sliding window constructs local neighborhood context for each position.

Finally, local feature autocorrelation calculation is performed. Let the similarity strength between the original features and the shifted features be $\;Corr\in {\mathbb{R}}^{C\times H\times W}$. The calculation process is shown in (18).

(18) $$Corr=\frac{1}{K^{2}}\sum_{i=1}^{K}\sum_{j=1}^{K}\left|{\left(W_{J}⊙W_{G}\right)}_{\ldots ,i,j}\right|$$

where ⊙ denotes the Hadamard product, and $\left|\cdot \right|$ represents the modulus operation.

To suppress weight imbalance caused by excessively high regional similarity strength due to strong scattering points, logarithmic compression is applied to equalize the distribution of similarity strength, thereby enhancing the visibility of microstructures. Subsequently, the normalized similarity strength is mapped to attention weights via the sigmoid function, and spatial weighting is performed on the original feature map. Finally, the sliding correlation attention-enhanced feature map $E_{c}\in {\mathbb{C}}^{C\times H\times W}$ is generated.

(19) $$\left\{\begin{array}{l} E_{c}=J⊙\sigma \left(\log\left(Corr\right)\right)\\ \sigma \left(\log\left(Corr\right)\right)=\frac{1}{1+\exp(-\log(Corr))} \end{array}\right.$$

#### 2.4.2. Subdomain Balanced Attention (SBA)

In ISAR echoes, amplitude information characterizes the distribution of target electromagnetic scattering intensity, while phase information exhibits high sensitivity to target micro-motions and subtle structural variations, providing complementary discriminative features. Most existing segmentation methods primarily process amplitude images alone, failing to fully establish a synergistic feature representation mechanism that enhances both amplitude and phase information. This limitation constrains the model’s ability to perceive and utilize key structures of complex targets.

**Figure 6:**
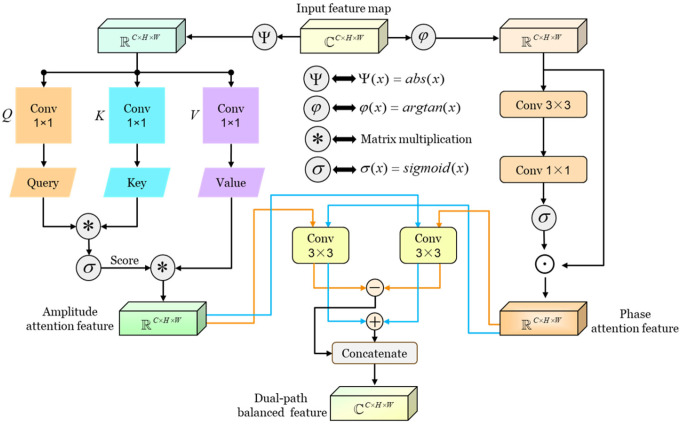
Structure of the SBA.

To address the aforementioned challenges, this paper proposes the SBA module. This module adaptively focuses on features in strong scattering regions through an amplitude attention mechanism and enhances structural perception of micro-motion components using a phase modulation attention mechanism. Finally, a dual-path coordination mechanism achieves constrained balance between amplitude and phase features, enabling physically consistent modeling of full-dimensional scattering characteristics in ISAR. The structure of SBA is shown in Figure 6.

First, compute the amplitude attention features. Let the input feature map of the SBA module be $E\in {\mathbb{C}}^{C\times H\times W}$, with its magnitude features Mag=abs(E), where $Mag\in {\mathbb{R}}^{C\times H\times W}$. Inspired by the self-attention mechanism in Transformers [25], this paper introduces three 1 × 1 convolutional layers to construct the Query Matrix $Q_{s}$, Key Matrix $K_{s}$, and Value Matrix $V_{s}$. The 1 × 1 convolutional layers preserve the original spatial structural relationships in ISAR semantic segmentation. This approach avoids the exponential increase in parameters associated with fixed-size weight matrices while enabling the modeling of scattering feature correlations along the channel dimension. The calculation process of the attention output is as follows.

(20) $$\left\{\begin{array}{l} q_{s}=Q_{s}(Mag)=Conv_{s1}(Mag)\\ k_{s}=K_{s}(Mag)=Conv_{s2}(Mag)\\ v_{s}=V_{s}(Mag)=Conv_{s3}(Mag) \end{array}\right.$$

The attention scores and magnitude attention features are subsequently computed as follows:

(21) $$\left\{\begin{array}{l} Score=\sigma (q_{s}*k_{s}^{T})\\ E_{m}=Score*v_{s} \end{array}\right.$$

where σ(⋅) denotes the Sigmoid function, and ∗ represents matrix multiplication.

Next, compute the phase attention features. The phase feature map Pha=argtan(Im(E)Re(E)), where $Pha\in {\mathbb{R}}^{C\times H\times W}$. The calculation process for the phase attention features is as follows:

(22) $$E_{p}=E⊙\sigma (Conv_{p1}(Conv_{p2}(Pha)))$$

By employing a dynamic weighted fusion strategy, a balance between amplitude and phase attention features is achieved, generating spatially discriminative subdomain attention-enhanced features $E_{b}\in {\mathbb{C}}^{C\times H\times W}$.

(23) $$\left\{\begin{array}{l} R_{b}=Conv_{b1}(E_{m})-Conv_{b2}(E_{P})\\ I_{b}=Conv_{b2}(E_{m})+Conv_{b1}(E_{P})\\ E_{b}=R_{b}+I_{b}j \end{array}\right.$$

### 2.5. Complex-Domain Decoder

The complex-domain decoder employs a Multi-branch Decoding Block (MDB) as its core architecture, utilizing parallel branches to fuse multi-scale complementary information at the same hierarchical level for refined feature representation. Simultaneously, it aggregates feature maps of corresponding scales from the encoder through skip connections, supplementing high-resolution spatial details. This design significantly suppresses boundary blurring effects during the progressive restoration of spatial resolution, thereby improving segmentation accuracy.

The input feature map of the decoder is denoted as $D\in {\mathbb{C}}^{C_{u}\times H_{u}\times W_{u}}$. Through multi-stage MDB processing, progressive upsampling is performed to restore the feature map resolution. Let $D_{i}\in {\mathbb{C}}^{C_{u}\times (H\times 2^{i})\times (W\times 2^{i})}\;(i=0,1,\ldots ,3)$, $D_{4}\in {\mathbb{C}}^{\frac{C_{u}}{2}\times (H\times 16)\times (W\times 16)}$, $D_{5}\in {\mathbb{C}}^{1\times (H\times 16)\times (W\times 16)}$, where $C_{u}$ represents the output channel number of MDB, and $D_{i}$ denotes the output feature map of the i-th MDB. $D_{1}$ and $D_{2}$ are connected via a 2 × 2 complex transposed convolutional layer to achieve feature reuse, integrating multi-scale contextual information to enhance segmentation accuracy. Finally, a 1 × 1 convolutional layer is applied to combine the features, which are then transformed through a Sigmoid function to output the pixel-wise semantic segmentation map.

(24) $$\left\{\begin{array}{l} D_{5}=R_{u}+I_{u}j\\ C=Conv(R_{u})+Conv(I_{u})\\ Mask=Sigmoid(C) \end{array}\right.$$

The MDB adopts a dual-branch feature fusion architecture, comprising a shallow feature branch and a deep feature branch. By designing parallel branch structures with varying depths, this module effectively integrates spatial information from different hierarchical levels, balancing local detail features with global semantic features, thereby significantly improving segmentation accuracy for both the main structure and boundaries of spacecraft.

The shallow feature branch consists of a 2 × 2 Complex Transposed Convolutional (CTC) layer followed by a CBN layer. Given the input feature map of the MDB as $D_{q}\in {\mathbb{C}}^{C_{q}\times H_{q}\times W_{q}}$, the output feature map of this branch can be expressed as:

(25) $$D_{shallow}=\xi (ConvTranspose(D_{q}))$$

where ξ(⋅) denotes CBN.

The deep feature branch consists of two 3 × 3 CTC layers, two CBN layers, and one CReLU layer. Given the same input feature map $D_{q}$, the output feature map of this branch can be expressed as:

(26) $$\left\{\begin{array}{l} D_{f}=\sigma (\xi (ConvTranspose(D_{q})))\\ D_{deep}=\xi (ConvTranspose(D_{f})) \end{array}\right.$$

where σ(⋅) denotes the CReLU function.

First, the dual-branch features are concatenated along the channel dimension to form the combined features $D_{dbl}$. Subsequently, this combined feature set is passed through a 1 × 1 complex convolutional layer for efficient fusion and dimensionality reduction, ultimately generating the output feature map $D_{out}$ of the MDB.

(27) $$\left\{\begin{array}{l} D_{dbl}=concatenate(D_{shallow},D_{deep})\\ D_{out}=Complex\text{\_}Conv(D_{dbl}) \end{array}\right.$$

## 3. Experimental Results

This paper designs a series of experiments to demonstrate the effectiveness of the proposed method. Section 3.1 introduces the ISAR target dataset used in the experiments; Section 3.2 defines the key parameter configurations and evaluation metrics. Based on this, Section 3.3 focuses on investigating the feasibility of the proposed AIL method for automatic labeling of ISAR data; Section 3.4 provides an in-depth analysis and validation of the effectiveness of each key module through ablation studies; finally, Section 3.5 conducts comparative experiments with representative algorithms of the same type, combining quantitative and qualitative analyses to prove the superior performance of OSSNet in ISAR echo semantic segmentation.

### 3.1. Datasets

**Figure 7:**
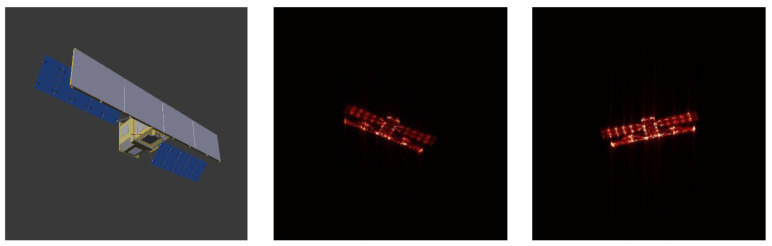
On the (**left**) is the CAD model diagram of the satellite, and in the (**middle**) and on the **(right**) are the images corresponding to the ISAR echoes.

This paper employs an ISAR dataset generated through ground-based radar model simulations. The simulation center frequency is 17 GHz, with a bandwidth of 2 GHz. The resolution of the simulated ISAR images is 0.075 m. Both the pitch and azimuth angles have a range of 60° for target imaging. The dataset comprises a total of 1793 sample pairs, with each pair consisting of raw ISAR echo data and its corresponding labels. The dataset was randomly partitioned into training and test sets at a 7:3 ratio using a fixed random seed (42) to ensure reproducibility. The input data are echo matrices of size 512 × 512, while the label data consist of segmentation masks generated by the AIL method. In Figure 7, the leftmost is the CAD model, and the rest are ISAR images corresponding to typical echo samples.

It is noteworthy that a domain gap exists between simulated and measured data in ISAR imaging. Simulations typically rely on ideal point-scattering models or high-frequency approximation methods, generating scattering centers with simplified and regular statistical characteristics in amplitude, position, and angular sensitivity, and coherent integration is performed under far-field and interference-free assumptions. In contrast, measured ISAR echoes inevitably contain nonlinear distortion, phase noise, and I/Q imbalance introduced by the radar system, as well as defocusing and elevated sidelobes caused by residual motion compensation errors. This gap necessitates appropriate domain adaptation when models trained on simulated data are applied to measured data.

### 3.2. Implementation Details and Evaluation Metrics

All experiments were conducted using the PyTorch 1.11.0 framework on a workstation equipped with a single NVIDIA GeForce RTX 3090 GPU (Nvidia, Santa Clara, CA, USA) with 24 GB of memory. During the training phase, the Adam optimizer was employed, with differentiated initial learning rates set according to parameter characteristics, where the learnable matrix parameters in the DAM had a learning rate of 1 × 10^−5^, and the parameters of other modules had a learning rate of 1 × 10^−4^. The weight decay is set to 1 × 10^−4^. Given the high foreground-background pixel imbalance caused by the small proportion of spacecraft targets in the masks, the BCEWithLogitsLoss function was selected as the loss function, with an increased positive sample weight to enhance the model’s learning capability for critical target regions. The model was trained with a batch size of 4 for 200 epochs. All experimental results are the average obtained under three different random seed settings. All models adopt Kaiming Uniform initialization and do not perform data augmentation to ensure fairness in comparison.

The relevant parameter settings for AIL are as follows: The Gaussian pyramid has a total of 3 layers, where the first layer is the original complex image, and the second and third layers are obtained by progressive downsampling, with weights of 1, 1/4, and 1/6 respectively. For morphological operations, rectangular structuring elements and disk structuring elements are used to perform closing first, followed by opening. In this paper, the threshold of AIL is set to 26. It is worth noting that the selection of this threshold relies on experience.

To comprehensively evaluate model performance, this paper adopts mean Intersection over Union (mIoU) and F1score as the core evaluation metrics. The IoU measures the overlap between the predicted segmentation and the ground truth, defined as the ratio of the intersection to the union of the predicted and true values. The mIoU is the average of the IoU values across all categories in the dataset. The F1score provides a comprehensive assessment by combining both recall and precision for each category, and the mF1 is the average of the F1scores across all categories. The specific mathematical definitions of these metrics are as follows:

(28) $$IoU=\frac{TP}{TP+FP+FN}$$

(29) $$mIoU=\frac{1}{N}\sum_{i=1}^{N}IoU_{i}$$

(30) $$\mathrm{Precision}=\frac{TP}{TP+FP}$$

(31) $$\mathrm{Recall}=\frac{TP}{TP+FN}$$

(32) $$F_{1}score=2\times \frac{\mathrm{Precision}\times \mathrm{Recall}}{\mathrm{Precision}+\mathrm{Recall}}$$

(33) $$mF_{1}==\frac{1}{N}\sum_{i=1}^{N}F_{1}score_{i}$$

where N=2 in this article denotes two categories (spacecraft and background) in the datasets we used; TP, FP, and FN denote the true positive, false positive, and false negative.

### 3.3. Validation of the AIL Annotation Method

**Figure 8:**
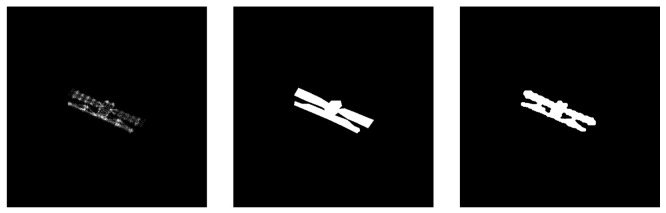
(**Left**) Original ISAR image; (**Middle**) Manually annotated mask; (**Right**) Automatically annotated mask by AIL.

To validate the effectiveness of the AIL method, images generated from ISAR echoes, manually annotated masks, and AIL-generated masks were uniformly converted into grayscale images for visual comparison, as shown in Figure 8. In Figure 8, the left panel shows the original ISAR image, the middle panel shows the manually annotated mask, and the right panel shows the automatically annotated mask generated by AIL. The visualization results demonstrate that while masks produced by traditional manual annotation exhibit higher regularity in geometric contours, they struggle to accurately reflect the inherent scattering point distribution characteristics of ISAR imagery. This limitation is particularly evident at critical connection regions of the spacecraft structure, where manual annotation fails to capture and represent subtle structural variations induced by complex electromagnetic scattering. In contrast, the AIL method demonstrates a superior capability to characterize these key regions in a manner more consistent with the underlying physical scattering mechanisms.

Although the evaluation of annotation mask quality still primarily relies on manual experience, to maintain consistency in quantitative metrics, this paper continues to adopt mIoU, F_1_score, Precision, and Recall as the quantitative benchmarks for comparing different annotation methods. Although the inherent uneven distribution of strong and weak scattering points and stripe interference in ISAR images may cause systematic deviations in these metric values, since both annotation methods are evaluated on the same dataset with comparable deviation levels, this evaluation framework remains effectively comparable.

As shown in Table 1, the AIL method outperforms traditional manual annotation across all four metrics: Recall increased by 2.72% (95.65 vs. 92.93), Precision increased by 1.57% (86.29 vs. 84.72), mIoU increased by 2.82% (83.75 vs. 80.93), and mF1 increased by 2.04% (90.40 vs. 88.36). Accurate perception of spacecraft structural boundaries is the prerequisite for obtaining high-quality masks. By constructing multi-scale complex gradient images to capture multi-resolution edge features and deeply modeling target scattering characteristics, AIL achieves higher structural consistency between labels and actual targets through gradient magnitude variations across different regions. Meanwhile, manual annotations often suffer from over-smoothing in weak scattering regions due to visual subjectivity, whereas the combination of complex gradient images and morphological processing effectively enhances edge continuity and geometric fidelity. Furthermore, the annotation method based on physical scattering characteristics demonstrates stronger stability and interpretability. Both visual comparisons and quantitative results fully validate the superiority of AIL’s discrimination mechanism based on physical thresholds of gradient magnitude in distinguishing complex structures, providing reliable training labels for subsequent deep learning-based semantic segmentation models.

**Figure 9:**
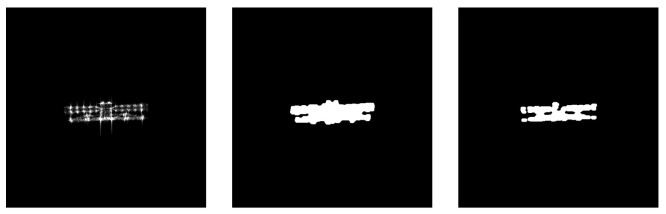
Failure case. (**Left**) Original ISAR image; (**Middle**) The mask when the AIL threshold is 16; (**Right**) The mask when the AIL threshold is 40.

Failure case analysis. To examine the sensitivity of the AIL method to the threshold parameter τ, we applied two extreme settings to a representative ISAR image, as shown in Figure 9. When τ is set too low (e.g., 16), the mask fails to isolate the spacecraft structure—sidelobe artifacts and weak scattering responses are retained, causing adjacent components (e.g., the body and solar panels) to merge into a single connected region. This results in a structurally ambiguous mask that lacks the necessary spatial separation for subsequent segmentation. Conversely, when τ is set too high (e.g., 40), legitimate but weaker gradient responses along the spacecraft edges and at component junctions are discarded. The resulting mask suffers from broken contours, internal cavities, and incomplete representation of slender parts such as solar panel edges. These observations indicate that a fixed threshold setting cannot optimally handle the inherent variations in scattering intensity within ISAR images, motivating future research toward adaptive thresholding strategies.

### 3.4. Ablation Study

**Figure 10:**
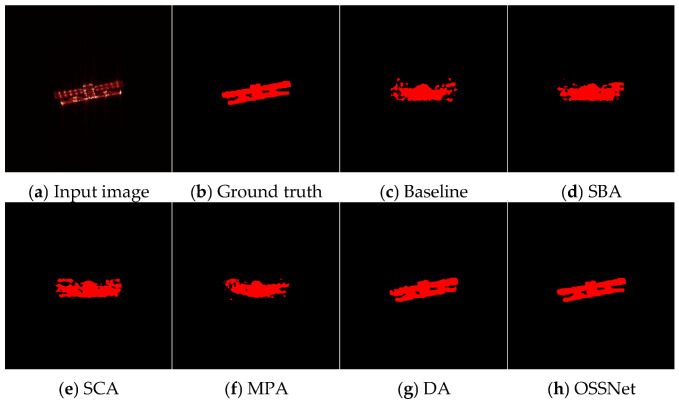
Results of ablation studies, where (**a**) is the image corresponding to the echo.

To systematically validate the effectiveness of key modules in OSSNet, this paper conducts ablation studies: using the basic OSSNet architecture without DAM, SCA, and SBA as the baseline model, the individual contributions of DAM, SBA, and SCA to segmentation performance are evaluated separately. Comprehensive quantitative evaluation results are presented in Table 2 with corresponding visualizations shown in Figure 10.

(1) DAM: Table 2 demonstrates that although the background region occupies a large proportion, resulting in consistently high IoU and F1scores for this category regardless of the DAM’s presence, the absence of the DAM module causes significant performance degradation in the most critical task of spacecraft main body segmentation. Compared to the baseline model, adding the DAM module alone increases the main body IoU by 13.82% (67.10 vs. 53.28) and the main body F1score by 10.79% (80.31 vs. 69.52). Regarding overall performance, the mIoU improves from 75.59% to 82.83%, and the mF1 improves from 84.23% to 89.79%. Combining these quantitative metrics with the visual comparison between Figure 10c,g leads to the following conclusion: DAM effectively bridges the data distribution gap between the echo domain and the mask domain by constructing an adaptive domain transformation function, resulting in segmentation outcomes with clear spacecraft contours, thereby laying a solid foundation for further improving segmentation accuracy.

Furthermore, compared to the complete OSSNet model, removing the DAM alone causes a severe performance drop: the main body IoU decreases by 29.98% (54.81 vs. 84.79), and the main body F1score decreases by 20.96% (70.81 vs. 91.77). In terms of overall performance, the mIoU drops from 92.13% to 76.43%, and the mF1 drops from 95.75% to 84.91%. Comparing the visualization results in Figure 10f,h leads to the following conclusion: The segmentation results after removing DAM show severely distorted spacecraft contours and numerous misclassified pixels, indicating that DAM provides an effective segmentation basis for subsequent modules, enabling them to learn the spatial distribution of the spacecraft structure within a feasible data domain and achieve precise segmentation. These results fully demonstrate that the baseline model and other modules struggle to establish accurate semantic mappings independently, highlighting the core role of DAM in achieving the critical transformation from the echo domain to the mask domain.

(2) SBA: As shown in Table 2, compared to the baseline model, adding the SBA module alone increased the main body IoU by 0.5% (53.78 vs. 53.28) and the main body F1score by 0.43% (69.95 vs. 69.52). In terms of overall performance, mIoU improved from 75.59% to 75.79%, and mF1 improved from 84.23% to 84.42%. A combined analysis of these quantitative metrics and the visual comparison between Figure 10c,d leads to the following conclusion: The segmentation results of the model incorporating SBA are more complete, indicating that SBA achieves more accurate main structure characterization through the joint modulation of amplitude and phase attention. However, comparison with the complete OSSNet model reveals that adding the SBA module alone provides limited improvement in segmentation quality, due to its insufficient capacity for fine-grained modeling of the spacecraft’s spatial structure.
(3) SCA: As shown in Table 2, compared to the baseline model, adding the SCA module alone increased the main body IoU by 1.12% (54.40 vs. 53.28) and the main body F1score by 0.95% (70.47 vs. 69.52). Regarding overall performance, mIoU improved from 75.59% to 76.16%, and mF1 improved from 84.23% to 84.71%. A combined analysis of these quantitative metrics and the visual comparison between Figure 10c,e leads to the following conclusions: The segmentation results of the model incorporating SCA are more focused, with reduced discrete misclassified points, indicating that SCA achieves a more concentrated main structure by computing spatial correlations. However, comparison with the complete OSSNet model reveals that adding the SCA module alone provides limited improvement in segmentation accuracy, as it cannot accurately distinguish fine structural boundaries within the original data distribution.
(4) MPA: Table 2 quantitatively reveals its significant contribution to segmentation accuracy. Compared with the baseline model, after adding MPA alone, the main body IoU increased by 1.53% (54.81 vs. 53.28), and the F1 score increased by 1.29% (70.81 vs. 69.52); in terms of overall performance, mIoU increased from 75.59% to 76.43%, and mF1 increased from 84.23% to 84.91%. The comparison between quantitative metrics and the visualization results of Figure 10c,f leads to the following conclusion: MPA, based on prior information of spacecraft structure and electromagnetic scattering characteristics, proposes the SCA and SBA modules, which can effectively guide the network to focus on key structural areas.

Meanwhile, compared with the complete OSSNet model, after removing DAM alone, the spacecraft main body IoU decreased by 17.69% (67.10 vs. 84.79), and the F1 score decreased by 11.46% (80.31 vs. 91.77); in terms of overall performance, mIoU decreased from 92.13% to 82.83%, and mF1 decreased from 95.75% to 89.79%. The comparison between the visualization results of Figure 10g,h leads to the following conclusion: Although the basic contour of the spacecraft is preserved, there are significant edge blurring and local misclassification phenomena. These results indicate that the baseline model combined with DAM can initially capture the main structural information of the spacecraft but struggles to achieve precise boundary localization; whereas MPA, through the synergistic mechanism of SCA and SBA, effectively guides the network to focus on the key features of the spacecraft main body and edges, significantly reducing misclassification and thereby substantially improving segmentation accuracy.

Based on the quantitative results in Table 2 and the visual analysis in Figure 10, it can be concluded that the proposed DAM primarily focuses on achieving a global transformation mapping from the echo domain to the semantic segmentation mask domain, serving as the fundamental basis for semantic parsing of raw echoes. In contrast, building upon the transformation provided by DAM, the MPA module guides the network to precisely focus on the main structure and edge details of the spacecraft through its spatial contextual and boundary perception mechanisms. The synergistic integration of DAM and MPA enables the model to achieve an optimal balance between overall segmentation quality and local detail preservation, ultimately realizing the best segmentation performance.

### 3.5. Comparison Experiment

To comprehensively evaluate the segmentation performance of OSSNet, this section conducts comparative experiments with nine advanced deep learning-based semantic segmentation methods, including FCN [26], DANet [27], PSPNet [28], DeepLabv3+ [29], U-Net [30], BiSeNet [31], Segformer [32], OffSeg [33], SegMAN [34], CL-NL-Unet [9] and CPGM [35]. Among them, FCN, DANet, PSPNet and DeepLabv3+ employ ResNet50 as their backbone network; for BiSeNet, BiSeNetv2 is adopted; for Segformer, MiT-B3 is used as the backbone network; for SegMAN and OffSeg, the corresponding default backbone configuration is used.

All datasets used in these experiments employ masks generated by the AIL method. It should be noted that the proposed OSSNet model in this paper is a semantic segmentation model that directly processes ISAR echo data. The comparative methods, in contrast, are all applied to segment-formed ISAR images, leveraging their proven success in image-based tasks. This difference highlighted the distinctiveness of OSSNet in fully utilizing original scattering information.

**Figure 11:**
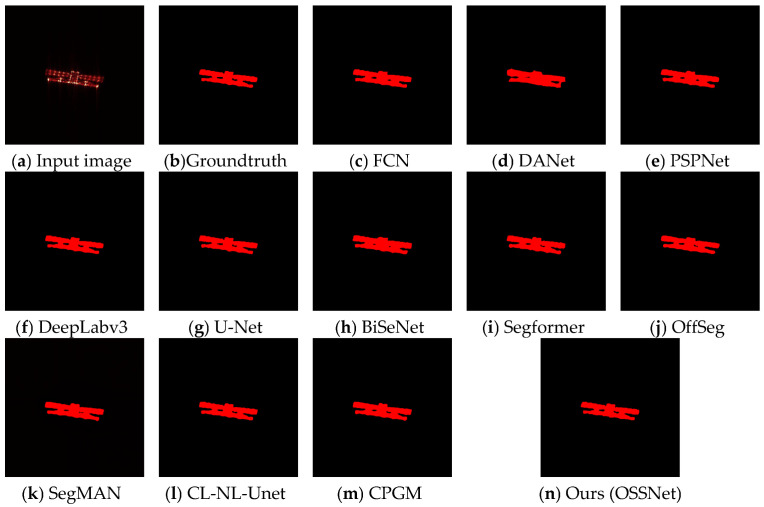
Comparison of segmentation results, where the input to OSSNet is the echo corresponding to (**a**).

To intuitively evaluate the performance advantages of OSSNet, Figure 11 provides a visual comparison of segmentation results from different methods on representative samples. For detailed observation, spacecraft targets are marked in red. Figure 11a displays the input ISAR image (used by comparative methods, while OSSNet uses its corresponding echoes), Figure 11b shows the ground truth, and Figure 11c–n sequentially present the segmentation results of FCN, DANet, PSPNet, DeepLabv3+, U-Net, BiSeNet, Segformer, OffSeg, SegMAN, CL-NL-Unet, CPGM and OSSNet. Observations reveal that constrained by inherent challenges in ISAR images—such as uneven scattering point distribution and complex scattering characteristics at key component junctions—other methods generally exhibit edge blurring and noise artifacts, as shown in Figure 11c–m. Among them, although DeepLabv3+ is relatively less affected, it still shows noticeable misclassification at solar panel connection points. In contrast, OSSNet’s segmentation results demonstrate superior edge clarity and structural integrity, effectively reducing such errors.

As shown in Table 3, OSSNet achieves a mIoU of 92.13% and a mF1 score of 95.75% on the test set, which are 1.62 and 0.96 percentage points higher than those of DeepLabv3+ (90.51% and 94.79%, respectively), the best-performing among the compared methods.

DANet and Segformer represent two widely adopted attention paradigms in semantic segmentation—dual attention and hierarchical self-attention, respectively—yet both underperform on ISAR data (spacecraft IoU: 65.43% and 73.20%). Their limitations stem from a shared design assumption: attention is computed globally over real-valued features without incorporating domain-specific physical structure. In DANet, position-wise attention normalizes pairwise similarities across all spatial locations via a global softmax. For natural images this aggregates semantic context effectively; for ISAR images, where strong scattering points can exceed weak structural edges in magnitude by an order of magnitude or more, the softmax collapses attention onto a few high-intensity positions and deprives boundary regions of gradient signals. Segformer’s multi-head self-attention partially alleviates this through hierarchical spatial reduction but inherits a fundamental limitation: it operates on amplitude-only images and thus discards the phase information. Neither method embeds inductive biases relevant to spacecraft—rigid-body symmetry, the distinct physical roles of amplitude and phase, or the spatial continuity of structural edges. OSSNet’s MPA framework addresses these gaps through two complementary mechanisms: SCA replaces global similarity with local autocorrelation, confining attention to a spatial neighborhood and preventing isolated scatterers from dominating; SBA separates amplitude and phase into dual processing paths, preserving the full scattering information that amplitude-only networks discard.

FCN achieves end-to-end pixel-level prediction by converting fully connected layers into convolutional layers, but its inherent downsampling operations lead to severe detail loss, resulting in coarse recovery of target contours in ISAR spacecraft semantic segmentation, which fails to meet the accuracy requirements for fine scattering point structure segmentation. PSPNet aggregates multi-scale contextual information from different regions through a pyramid pooling module to enhance global perception, but its multi-level pooling operations disrupt the structural integrity of key scattering points in ISAR data and introduce background noise during feature fusion, causing a significant decline in the segmentation performance of weak scattering targets. DeepLabv3+ combines multi-scale atrous convolutions with an encoder–decoder structure to balance contextual semantics and detail recovery, but its complex ASPP module and decoder tend to over-smooth critical scattering point texture features. UNet achieves precise local feature localization through a symmetric encoder–decoder architecture and skip connections, but its upsampling process struggles to reconstruct the highly discrete distribution characteristics of scattering points in ISAR data, leading to fragmentation in weak scattering regions. BiSeNet enables fast semantic segmentation at very low computational cost by constructing a dual-branch parallel structure with spatial and contextual paths, but its dual-branch feature fusion strategy fails to reconcile feature conflicts between sparse scattering points and dense backgrounds in ISAR data, causing key target features to be diluted during fusion and resulting in low distinction in segmentation boundaries. OffSeg improves feature alignment by learning spatial offsets, which refines the correspondence between decoder features and class representations. However, its offset prediction branch relies on continuous image gradients to estimate accurate displacement vectors. In ISAR images, scattering intensity varies abruptly and discontinuously across structural boundaries. Under this condition, the learned offsets become unreliable, leading to jagged or misaligned segmentation boundaries. SegMAN integrates state space models (SS2D) with neighborhood attention to simultaneously model global context and local details. However, SS2D compresses global context per channel into a fixed-dimensional hidden state, inherently discarding fine-grained spatial information. In ISAR data, where weak-scattering structural edges carry critical boundary information at low intensity, this compression risks suppressing these subtle yet essential responses. CL-NL-Unet introduces non-local self-attention and contrastive learning into ISAR segmentation. Its global receptive field helps capture the structural symmetry of space targets. However, the softmax normalization in the non-local operation tends to allow a few strong scattering points to monopolize the attention weights, thereby suppressing responses from weak scattering edges and impairing the integrity of the spacecraft main body. CPGNet employs category-pose joint guidance to explicitly modulate semantic features, with the intention of enhancing the model’s perception of target morphology. In our dataset, however, only a single spacecraft category exists. As a result, the fine-grained category partitioning strategy degenerates into ordinary binary segmentation, and the class-level prior provides little additional constraint.

The performance advantages of OSSNet primarily stem from its comprehensive exploitation of the original scattering characteristics in ISAR echoes and the effective guidance provided by spacecraft structural priors: the DAM achieves adaptive mapping from echo signals to semantic features, effectively mitigating data distribution discrepancies and enabling more efficient semantic feature representation. Simultaneously, by incorporating spacecraft structural priors, the MPA module guides the network to focus on critical features, enhancing the discriminative capability for spacecraft structures and edges. It is noteworthy that although the experimental dataset contains a large proportion of background regions, resulting in excellent IoU and F1score performance for the background category across all methods, OSSNet still achieves the highest background segmentation accuracy (IoU: 99.48%, F1score: 99.74%). More importantly, significant performance variations are observed among different methods in the critical task of spacecraft main body segmentation, reflecting the challenges posed by the complex structure of spacecraft. OSSNet achieves optimal performance in this key task (IoU: 84.79%, F1score: 91.77%), highlighting the robust perception capability of its physics-based semantic representation for complex scattering structures. Both qualitative comparisons and quantitative analysis jointly demonstrate that OSSNet, through its unique model design, excels in modeling and integrating the physical scattering characteristics inherent in ISAR echoes, thereby exhibiting significant advantages in ISAR semantic segmentation tasks.

As shown in Table 4, the proposed OSSNet achieves the highest mIoU and mF1 with 137.47 M parameters and 199.28 G FLOPs. Although OSSNet has the largest parameter count among all methods, its FLOPs remain moderate—substantially lower than U-Net (386.56 G) and CL-NL-Unet (565.12 G), and only slightly higher than Segformer (142.90 G) and DeepLabv3+ (138.90 G). The higher parameter count primarily stems from the multi-branch encoder (MEB), the SCA and SBA modules.

## 4. Conclusions

This paper proposes an OSS framework for on-orbit spacecraft segmentation using ISAR echoes. Its core comprises AIL and OSSNet. AIL efficiently generates high-quality semantic masks based on the inherent scattering characteristics of complex-valued ISAR images, reducing the cost of manual annotation. OSSNet employs an encoder–decoder architecture to directly process ISAR echo data, fully preserving original physical information while avoiding information loss inherent in the imaging process. The key innovations include: (1) a DAM that achieves adaptive alignment from the echo domain to the semantic domain through orthogonal transformation constraints, effectively resolving numerical domain mismatch; (2) a MPA integrating SCA and SBA modules, which deeply incorporates spacecraft structural priors with ISAR scattering characteristics to guide the network in precisely focusing on main structures and edge features from multiple perspectives, significantly improving segmentation accuracy and enhancing model interpretability. The decoder adopts a multi-branch structure without attention mechanisms, reconstructing high-quality segmentation maps through hierarchical feature fusion. Simulation experiments validate the effectiveness and superiority of the OSS framework. Beyond quantitative performance, the OSS framework offers interpretability through its physically motivated module design. SCA’s local autocorrelation operator explicitly encodes the Wiener-Khinchin relationship between structural periodicity and correlation peaks, meaning its attention weights are interpretable as detectors of geometrically regular spacecraft components. SBA’s dual-path architecture mirrors the physical roles of amplitude and phase in radar scattering, allowing its behavior to be understood as a learned balance between these complementary information channels. The ablation study (Table 2) provides additional functional interpretability by isolating each module’s contribution: DAM accounts for the echo-to-semantic mapping, while MPA refines structural boundary perception.

Limitations. 1. The design of the AIL method gives it an advantage in binary semantic segmentation mask annotation, whereas its application to multi-class semantic segmentation mask annotation is still being explored. 2. All experiments are conducted on simulated ISAR data generated from ground-based radar models. Validation on real measured ISAR data is necessary before practical deployment. 3. OSSNet directly processes complex-valued ISAR echoes, which contain both amplitude and phase information, whereas all compared methods operate on amplitude-only images formed after imaging. Consequently, a portion of OSSNet’s performance advantage is attributable to the richer input representation rather than the architectural design alone. Future work should extend the comparison to methods that also exploit complex-valued inputs, once such implementations become publicly available.

Several directions merit future investigation. First, validation on field-measured ISAR data is essential to assess the framework’s robustness to real-world noise characteristics and target variability that may not be fully captured by simulation. Second, cross-domain generalization—across different radar parameters, frequency bands, and target types—should be systematically evaluated to understand the transferability of the learned representations. Third, a detailed computational efficiency analysis, including inference latency, memory footprint, and throughput under different hardware constraints, is needed to determine suitability for time-sensitive space situational awareness applications.

## Figures and Tables

**Table 1:** Comparison between AIL and traditional manual annotation methods. The traditional methods are implemented by researchers in this field.

Method	Recall (%)	Precision (%)	mIoU (%)	mF_1_ (%)
Traditional	92.93	84.72	80.93	88.36
**Ours (AIL)**	**95.65**	**86.29**	**83.75**	**90.40**

**Table 2:** Results of ablation studies.

**Baseline**	**SCA**	**SBA**	**DAM**	**IoU per Class (%)**		**mIoU (%)**	**F_1_score per Class (%)**		**mF_1_ (%)**
				**Background**	**Spacecraft**		**Background**	**Spacecraft**	
				97.90	53.28	75.59	98.94	69.52	84.23
		√		97.80	53.78	75.79	98.89	69.95	84.42
	√			97.91	54.40	76.16	98.95	70.47	84.71
	√	√		98.06	54.81	76.43	99.02	70.81	84.91
			√	98.57	67.10	82.83	99.28	80.31	89.79
	√	√	√	99.48	84.79	92.13	99.74	91.77	95.75

**Table 3:** Comparison of segmentation results between OSSNet and state-of-the-art methods.

Method	IoU per Class (%)		mIoU (%)	F_1_score per Class (%)		mF_1_ (%)
	Background	Spacecraft		Background	Spacecraft	
FCN	99.27	80.01	89.64	99.63	88.89	94.26
DANet	98.46	65.43	81.95	99.22	79.10	89.16
PSPNet	99.21	78.58	88.90	99.60	88.01	93.80
DeepLabv3+	99.35	81.68	90.51	99.67	89.91	94.79
U-Net	99.29	80.48	89.89	99.65	89.19	94.42
BiSeNet	99.15	77.38	88.26	99.57	87.25	93.41
Segformer	98.93	73.20	86.07	99.46	84.53	92.00
OffSeg	99.31	80.92	90.11	99.65	89.46	94.56
SegMAN	99.35	81.53	90.44	99.67	89.85	94.76
CL-NL-Unet	99.11	75.94	87.53	99.54	86.43	92.99
CPGM	99.32	79.48	89.40	99.63	88.49	94.06
**Ours**	**99.48**	**84.79**	**92.13**	**99.74**	**91.77**	**95.75**

**Table 4:** Comparison of Params and FLOPs among different methods.

Method	Params (G)	FLOPs (G)	mIoU (%)	mF_1_ (%)
FCN	32.95	277.66	89.64	94.26
DANet	49.48	116.68	81.95	89.16
PSPNet	8.83	52.38	88.90	93.80
DeepLabv3+	40.35	138.90	90.51	94.79
U-Net	31.04	386.56	89.89	94.42
BiSeNet	3.61	25.72	88.26	93.41
Segformer	47.22	142.90	86.07	92.00
OffSeg	18.29	63.66	90.11	94.56
SegMAN	42.76	69.68	90.44	94.76
CL-NL-Unet	27.67	565.12	87.53	92.99
CPGM	13.74	50.06	89.40	94.06
**Ours**	**137.47**	**199.28**	**92.13**	**95.75**

## Data Availability

The data that support the findings of this study are available from the corresponding author upon reasonable request.

## Abbreviations

The following abbreviations are used in this manuscript:

ISAR	Inverse Synthetic Aperture Radar
OSS	One-Stop Segmentation
DAM	Domain Alignment Module
SBA	Subdomain Balanced Attention
SCA	Sliding Correlation Attention
MPA	Multi-Perspective Attention
